# Depressive Symptoms Feature-Based Machine Learning Approach to Predicting Depression Using Smartphone

**DOI:** 10.3390/healthcare10071189

**Published:** 2022-06-24

**Authors:** Juyoung Hong, Jiwon Kim, Sunmi Kim, Jaewon Oh, Deokjong Lee, San Lee, Jinsun Uh, Juhong Yoon, Yukyung Choi

**Affiliations:** 1 Department of Intelligent Mechatronics Engineering, Sejong University, Seoul 05006, Korea; jyhong@rcv.sejong.ac.kr (J.H.); jwkim@rcv.sejong.ac.kr (J.K.); 2 Department of Psychiatry, Yongin Severance Hospital, Yonsei University College of Medicine, Yongin 16995, Korea; sunmik204@yuhs.ac (S.K.); jaewonoh@yuhs.ac (J.O.); pangelt@yuhs.ac (D.L.); sanlee@yonsei.ac.kr (S.L.); 3 Department of Psychiatry, The Institute of Behavioral Science in Medicine, Yonsei University College of Medicine, Seoul 03722, Korea; 4 Mobigen Co., 128, Beobwon-ro, Songpa-Gu, Seoul 05854, Korea; suh@mobigen.com; 5 Korea Electronics Technology Institute, Seongnam-si 13509, Korea; jhyoon@keti.re.kr

**Keywords:** depressive symptoms feature, depression prediction, machine learning, smartphone

## Abstract

With the impact of the COVID-19 pandemic, the number of patients suffering from depression is rising around the world. It is important to diagnose depression early so that it may be treated as soon as possible. The self-response questionnaire, which has been used to diagnose depression in hospitals, is impractical since it requires active patient engagement. Therefore, it is vital to have a system that predicts depression automatically and recommends treatment. In this paper, we propose a smartphone-based depression prediction system. In addition, we propose depressive features based on multimodal sensor data for predicting depressive mood. The multimodal depressive features were designed based on depression symptoms defined in the Diagnostic and Statistical Manual of Mental Disorders (DSM-5). The proposed system comprises a “Mental Health Protector” application that collects data from smartphones and a big data-based cloud platform that processes large amounts of data. We recruited 106 mental patients and collected smartphone sensor data and self-reported questionnaires from their smartphones using the proposed system. Finally, we evaluated the performance of the proposed system’s prediction of depression. As the test dataset, 27 out of 106 participants were selected randomly. The proposed system showed 76.92% on an f1-score for 16 patients with depression disease, and in particular, 15 patients, 93.75%, were successfully predicted. Unlike previous studies, the proposed method has high adaptability in that it uses only smartphones and has a distinction of evaluating prediction accuracy based on the diagnosis.

## 1. Introduction

The COVID-19 pandemic has caused problems in a variety of aspects of daily living. It caused economic difficulties, decreased external activities, disconnection of relationships, and fear of infection, which resulted in many people suffering from anxiety or depression [1,2]. If left untreated, depression can lead to serious consequences, such as suicide. Studies have shown that people with mental disorders, such as depression and schizophrenia, are particularly vulnerable to COVID-19 [3]. In addition, the U.S. Centers for Disease Control and Prevention (CDC) defined people with mental problems, including depression, as a group at high risk for COVID-19 [4]. It is important to diagnose and treat depression as soon as possible so that it does not result in severe consequences. Self-report questionnaires are one of the most common tools for identifying depression. However, this strategy is impractical, since patients must actively assess if they are suffering symptoms of depression on their own. Consequently, a system that automatically identifies depression without user intervention is required.

Recently, mobile device research has been conducted to identify depression automatically. In real-time, mobile devices such as smartphones and wearable devices generate a variety of data. Mobile devices may generate and transmit data at any time and place. The user’s behavior can be inferred by designing a derived feature with a high correlation between the generated data and the behavior. Consequently, it is feasible to automatically estimate a user’s behavior without regard to time or place when utilizing a mobile device. In this paper, depression is predicted based on the relationship between mobile device data and representative depressive symptoms. The Diagnostic and Statistical Manual of Mental Disorders, Fifth Edition (DSM-5) [5] identifies nine symptoms of major depressive disorders. We aim to predict automated behavior based on the presence of severe depression disorder symptoms using smartphone data. Consequently, detecting the user’s behavior type is important for automated assessment.

Wang et al. [6] proposed a system for collecting mobile sensor data and self-report questionnaire data from smartphones for 48 college students, showing significant prediction of depression through the system. In addition, based on the prediction of depression through smartphones, many studies have proposed various derived feature design methods related to depression. Colbaugh et al. [7] predicted depression by measuring the amount of activity through GPS and WiFi data. Ware et al. [8] used metadata obtained from an institution’s WiFi infrastructure beyond GPS and WiFi data for college students to predict depression. In addition, existing studies [9,10] confirmed that the derived features related to depression through sensor data showed a correlation. Furthermore, many studies have used additional devices such as smartwatches beyond smartphones to collect more sophisticated and diverse sensor data. Narziev et al. [11] predicted depression by estimating five symptoms from data collected from smartphones and smartwatches for four weeks in 20 ordinary people. Wang et al. [12] predicted depression by designing features for poor concentration and depressed mood using smartphones and smartwatches for 83 college students. In summary, related studies design derived features associated with DSM-5-defined depressed symptoms using smartphone data. However, existing papers often have a small number of recruiters for gathered datasets [13,14]. Moreover, since datasets are collected for ordinary people, it often has a “data imbalance” problem in which participants with depression have extremely few [8]. In addition, research involving extra devices, such as wearable devices, is difficult to implement in regular activities.

In this paper, a system for predicting depression using only smartphones was proposed. We design derived features for symptoms defined in DSM-5 using data generated from smartphones and propose a new design method for deriving features for new deep learning-based expression features. The proposed system consists of a big data-based cloud platform that can develop a “Mental Health Protector” application that includes the ability to collect datasets and process large amounts of data. Smartphone sensor data and self-reported questionnaires of 106 participants who visited psychiatrists are collected and evaluated to verify multimodal derived features and depression prediction systems for the proposed depression prediction system. The depression prediction system we propose has high versatility because it uses only smartphones without the need for additional equipment. In addition, by comparing medical diagnosis and prediction, we confirm that our proposed derived features and questionnaires are accurate and effective in detecting patients with depression.

The aim of this paper is to predict depression automatically using smartphones. The contributions of this paper are as follows:

- We obtained datasets from mental patients using smartphones. Since we collected data for psychiatric patients, not ordinary people, there was no difference in the number of depressed and non-depressed patients. Therefore, balanced data were collected to represent the features of each group.
- We only use smartphones to predict depression. In contrast to methods that need extra wearable devices, the proposed system is easily accessible to many people.
- We propose a multimodal-based automatic depression prediction system. In the proposed system, features extracted from images using deep learning are newly designed. It was found that multimodal-based features enhance performance complementarily.

## 2. Materials and Methods

**Figure 1:**
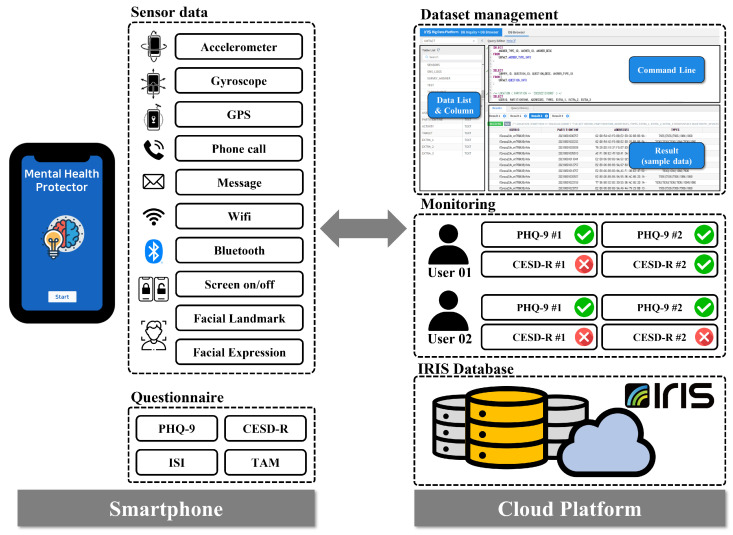
Proposed Architecture. This illustrates the architecture of the proposed depression prediction system. The “Mental Health Protector” application installed on the participant’s smartphone collects multimodal-based passive sensor data and active data, consisting of survey results. The collected data are transmitted to a cloud-based platform for data processing and storage. The participant’s depression is predicted and monitored using the data transmitted to IRIS.

The proposed system collected multimodal smartphone data from 209 Korean mental patients between 19 August and 2 October 2021. However, out of a total of 209 participants who stated their intention to participate in the experiment, a total of 103 participants were eliminated for various reasons, such as quitting during the study, missing sensor data, and failing to complete a self-reported questionnaire. Consequently, we conducted the study using data from 106 participants. We developed the Android-based “Mental Health Protector” application to collect various data from smartphones. The application was used to collect passive sensor data including GPS, Screen On/Off, Call logs, and SMS logs, Activity Transition, Facial expression features, etc., and survey response data including the Patient Health Questionnaire-9 (PHQ-9) [15], Center for Epidemiologic Studies Depression Scale-Revised (CESD-R) [16], The Insomnia Severity Index (ISI), and Technology Acceptance Model (smartphone use, app use). In the proposed system, ’IRIS’, a cloud-based big data platform, was used to collect and store big data. Finally, the proposed derived features were extracted from the collected data to predict depression in the user. The proposed system is represented in Figure 1.

### 2.1. Data Collection

#### 2.1.1. Research Procedure

**Figure 2:**
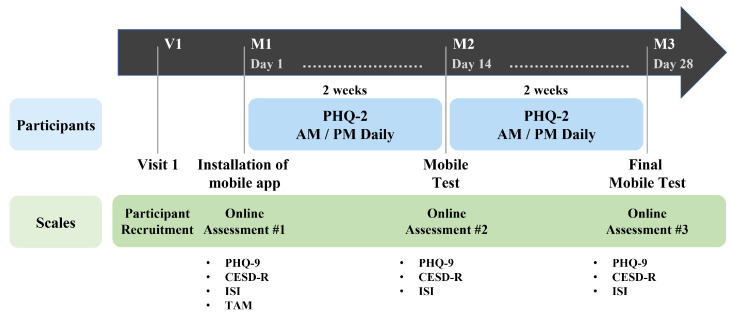
Data Collection Procedure. Among psychiatric outpatients, the data collection procedure was informed to users who agree to participate in the study (V1). When the application installation was completed, data were collected for two weeks (M1–M2) after conducting a survey related to the user’s demographic information and mental health (M1). After the first data collection period, a middle test (M2) was conducted, and the second data collection (M2–M3) was performed for two weeks again. When the second data collection is completed, the final test (M3) was conducted, and the participant’s role in the study ended.

Our study was conducted for four weeks on psychiatric outpatients who consented to the study’s purpose and data collecting. The collected data are “passive data” collected from multimodal sensors on smartphones and “active data” obtained from responses to self-reported questionnaires. At the stage of the hospital visit (V1), participants who consented to participate in the research signed the experiment consented form, and a basic survey was administered. In the basic survey (M1), participants installed the “Mental Health Protector” application on their smartphones, where they responded to demographic information and several mental health surveys. The mental health surveys include Patient Health Questionnaire-9 (PHQ-9), Center for Epidemiological Studies Depression Scale-Revised (CESD-R), Insomnia Severity Index (ISI), and Technology Acceptance Model Questionnaire (TAM). When the basic survey was completed, the first data collection (M1–M2) began. During the first data collecting period of two weeks, passive data were collected automatically through the application. A facial image was obtained from the camera at one-week intervals depending on the basic survey. The participants were then requested to respond to the Patient Health Questionnaire-2 (PHQ-2) twice daily, in the morning and afternoon. The data-collecting period was set at two weeks given that the mental health survey is based on behavior during the previous two weeks. When the collection of primary data was completed, a middle test (M2) was conducted. Using the application, participants were re-assessed using the PHQ-9, CESD-R, and ISI for the middle test. When the middle test was completed, the second data collection (M2-M3) was conducted during the same two-week period as the first data collection. When the second data collection was completed, the final test (M3) was conducted using the same setup as employed in the middle test, and the experiment was concluded. Figure 2 illustrates our research procedure.

#### 2.1.2. Mental Health Protector Application

The “Mental Health Protector” application is developed to store sensor data and perform self-reported questionnaires for participants. The developed application reads and saves data generated continuously by smartphone sensors. All sensor data are automatically saved without any action or command from the participant. The application can access the sensor and stores the sensor data in the background. Therefore, sensor data are collected with minimal participant intervention. The collected sensor data are Accelerometer, Gyroscope, Global Positioning System (GPS), Call logs, Short Message Service (SMS) logs, Wi-Fi, Bluetooth, and Screen on/off. In addition to sensor data, facial images based on the image domain are also collected. Facial images are used to obtain Facial Landmarks and Facial Expression Features. The application contains a model for transforming facial images into facial landmarks and facial expression features.

The “Mental Health Protector” application is developed for Android. The proposed application utilizes Android Studio 1.4 or a later version, and the API requires 16 (Jelly Bean) or a later version. The application’s User Interface (UI) was created using Vue.js. Vue.js is a JavaScript framework for developing UIs. Firebase Cloud Messaging (FCM) was used to communicate metadata from the smartphone application to the server. Using FCM, data may be handled regardless of the app’s foreground/background status.

**Figure 3:**
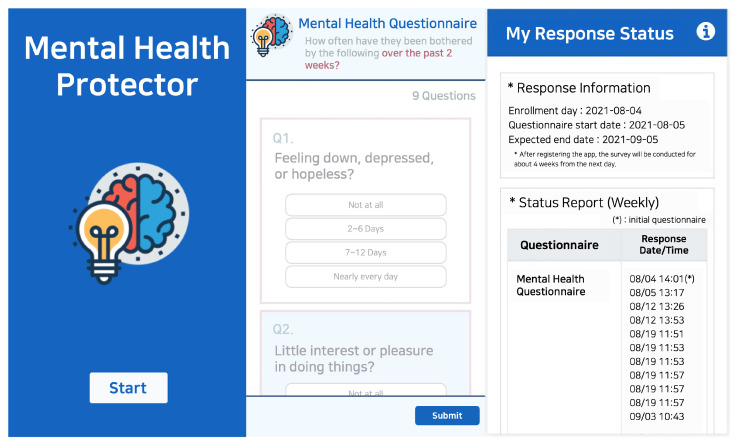
User Interface of “Mental Health Protector” application. The image shows a part of the data collection application titled “Mental Health Protector” execution screen. The image on the left depicts the initial execution screen, the image in the center depicts the screen that responds to the survey, and the image on the right represents the screen that can check the response status.

The application requires participants to respond to mental health-related questionnaires. Mental health-related surveys that participants must respond to are The Patient Health Questionaire-9 (PHQ-9), Center for Epidemiological Studies Depression Scale-Revised (CESD-R), The Insomnia Severity Index (ISI), and the Technology Acceptance Model (smartphone use, app use). The proposed system assesses the severity of depression using two self-reported questionnaires (PHQ-9 and CESD-R). Therefore, it is possible to strengthen the dependability of the participants’ responses and cross-verify their depressive feelings. The application sends a notification to the participant’s smartphone at the designated period for each questionnaire, allowing the participant to respond. If the participant does not respond to the survey, the application returns the notification to the participant’s smartphone. Consequently, the “Mental Health Protector” application stores sensor data and surveys answer data. In addition, the application allows participants to view their response statistics. On the “my status” tab, participants access their application registration details and the status of their survey replies. The “Mental Health Protector Chart” provides details from the completed questionnaire. Figure 3 depicts the execution screen of the “Mental Health Protector”.

The proposed system utilizes “IRIS”, a cloud-based big data platform, to store data from all participants’ smartphones. IRIS is a distributed architecture database that can process large-scale time-series data quickly and integrally supports the collection, storage, processing, distributed processing, analysis, visualization, and sharing of big data. The “Mental Health Protector” application obtains data from the smartphone’s multimodal sensors. The smartphone stores the received data in the form of a CSV file according to a pre-determined table. Table 1 represents the predefined data table. Once each day, the smartphone transmits the stored data to IRIS. Given the large scale of the multimodal sensor data, the proposed system only transmits sensor data to IRIS when the smartphone is connected to WiFi. In contrast, the questionnaire answer data are transmitted to IRIS, regardless of the WiFi connection status. Through the IRIS platform, collected data may be accessed at any time.

#### 2.1.3. User Characteristics

In this paper, we recruited 209 participants from psychiatric outpatients. According to the collection procedure, the data of all participants were collected for four weeks. However, we excluded certain participants from the depression prediction experiment. The experiment could not be conducted because there was an anomaly in the data of the participants. Specifically, since the proposed system uses the participants’ survey answer as depressive state, the response data of the participant are essential. However, 67 participants did not respond to all questionnaires. In addition, a significant amount of sensor data for 23 participants are missing; thus, the depression-related features are not extracted. Missing sensor data are caused by the termination of smartphones, lack of battery in smartphones, sensor failure, failure to transmit sensor data, etc. Nine participants indicated quitting in the middle of the study. Consequently, 106 participant data were used in this study after 103 participants were eliminated.

For the gender distribution among the participants, 43/106 (40.57%) are male and 63/106 (59.43%) are female. For the participants’ year of birth distribution, 4/106 (3.77%) were born in the period of 1960 to 1964, 6/106 (5.66%) were born in 1965 to 1969, 13/106 (12.26%) were born in 1970 to 1974, 17/106 (16.04%) were born in 1975 to 1979, 14/106 (13.21%) were born in 1980 to 1984, 7/106 (6.60%) were born in 1985 to 1989, 14/106 (13.21%) were born in 1990 to 1994, 15/106 (14.15%) were born in 1995 to 1999, and 16/106 (15.09%) were born in 2000 to 2004. According to Kroenke [15], the severity of depression as assessed by the PHQ-9 depression score is categorized as normal (0–4), minimum (5–9), mild (10–14), moderate (15–19), and severe (20–27). According to Lee [17], the CESD-R depression score was classified as Depressed or Non-Depressed based on a cut-point of 13 that reflected the sociocultural background of Korea. Eighty-four participants had depressed moods based on the CESD-R score, while 22 participants did not. Table 2 provides the participants’ demographics.

#### 2.1.4. Privacy Consideration

In this paper, multimodal sensor data are collected from participants. However, the collected data contain personal information; therefore, we designed the system to secure personally identifiable information during data collection and processing. To avoid the identification of individuals through the collected data, the data of all participants are anonymized by assigning a random hash ID to each smartphone when the application is installed. All sensor data are transmitted to the IRIS system using the hash ID. Consequently, the proposed system is unable to identify the participant based on the stored sensor data. The proposed system does not collect sensitive information. SMS logs and Call logs data are logs of text messages or phone calls. Therefore, the proposed system does not collect textual content, call conversation, or the phone numbers of other people. The only data obtained are whether the event is received or sent and when it occurred. Facial images are used to obtain “Facial Expression Features” and “Facial Landmarks”. The facial images captured from the camera are not transmitted to the IRIS system. A model inside the application transforms the face image into facial features. Both “Facial Expression Features” and “Facial Landmark” are translated to quantitative values from facial images within the application. Therefore, in the proposed system, the facial image is not sent to the server, but only the numerical feature is transmitted. In addition, it is impossible to convert the server-stored feature back into the face image.

### 2.2. Multimodal Feature Extraction for Depression Prediction

**Figure 4:**
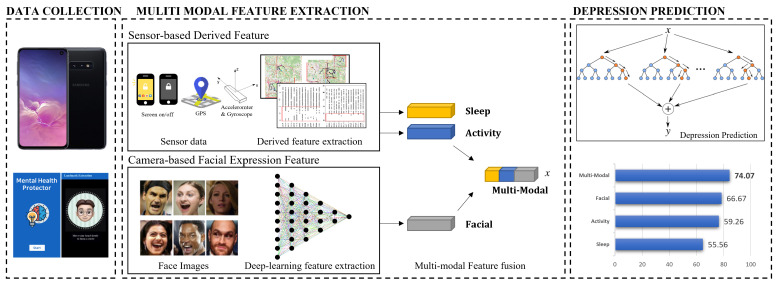
Overview of the Smartphone-Based Depressed Mood Prediction System. The system for predicting depression consists of three parts (Data Collection, Multimodal Feature Extraction, and Depression Prediction). In the Data Collection, the “Mental Health Protector” program collects multimodal sensor data from smartphone. In the Multimodal Feature Extraction, derived features are extracted from the collected data. In the Depression Prediction, a machine learning-based classifier is used to the derived features to predict the user’s depression.

A smartphone generates a variety of data, including sensor data and user-response data. Existing works [18,19] have found that there is a relationship between participants’ behaviors and behavioral features derived from smartphone data. Motivated by these studies, we design the behavioral features associated with major depressive disorder symptoms to predict participants’ depression. According to DSM-5 [5] published by the American Psychiatric Association, the symptoms of major depressive disorder are depressed mood, diminished interest and pleasure in activities, fatigue, restlessness, sleep change, weight change, diminished ability to concentrate, feelings of worthlessness, and thoughts of death and suicide. Since behavior-related symptoms can be estimated from smartphone data, we focus on two behavior-related symptoms: “diminished interest and pleasure in activities” and “sleep change”. Furthermore, novel derived features are proposed to be extracted from the image domain data. Facial expression characteristics were designed to detect depression from facial images. As a result, the proposed system proposes derived features based on multimodal data. Figure 4 provides a summary of the multimodal features and depressive state prediction algorithms.

#### 2.2.1. Derived Feature Based on Passive Sensor Data

In this study, derived features related to sleep and physical activity are designed based on smartphone sensor data. The derived features for sleep are “amount of sleep” and “quality of sleep”. Based on Min et al. [20], who predicted the sleep pattern using the screen on/off pattern, the derived features for “amount of sleep” are designed. Therefore, the sleep feature is estimated as the longest amount of time of the day when the smartphone screen is off. If the estimated time of sleep exceeds 24 h or if the period between 12 p.m. and 18 p.m. is estimated to be the sleep time, the screen on/off data are omitted and eliminated from the sleep time. The derived feature for “quality of sleep” is designed based on screen on/off data from Sano et al. [21], who show a correlation between smartphone usage and quality of sleep. Usage time is the time when the smartphone’s screen is on. Seven statistical features are extracted for each of the estimated sleep times and smartphone usage. Statistical features include maximum, minimum, average, standard deviation, first quartile, second quartile, and third quartile. Consequently, a total of 14 derived features for sleep are designed utilizing screen on/off data.

The derived features for physical activity include “location variance”, “entropy”, and “the amount of physical activity per day”. In Saeb’s study [22], “location” and “entropy” are proposed as derived features, and it is demonstrated that they were highly correlated with depression. In addition, these derived features are commonly utilized in other smartphones sensor-based depression prediction studies. The proposed system also uses the same derived features associated with physical activity. Therefore, derived features from GPS data are extracted using DBSCAN (density-based spatial clustering of applications with noise) clustering. The application extracts the amount of daily physical activity using the Google Activity Recognition Transition API. The Google Activity Recognition Transition API recognizes a total of eight behaviors using gyroscope and accelerometer sensors: vehicle, cycling, walking, running, walking or running, stationary, tilting, and unknown. Among the eight classes, the proposed system calculates the average daily physical activity time using stationary, running, and walking, which represent physical activity excluding bicycles, which can be confused with vehicle.

#### 2.2.2. Facial Expression Feature Based on Camera

**Figure 5:**
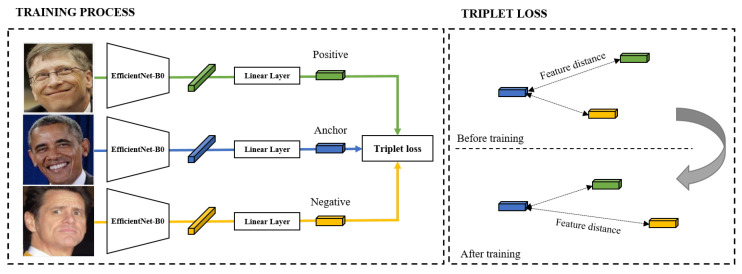
Architecture for Facial Expression Feature. This represents the training process and loss for deep learning-based facial expression feature design.

Based on Zhou [23], we design derived features from facial images for depression prediction. Facial expression features proposed by Google AI [24] are commonly applied in expression-based applications. The proposed method constructs a network with EfficientNet B0 as the backbone for the generation of latent variables from facial images. Triplet loss-based metric learning is utilized in order to train the model. We use a large-scale face dataset, Facial Expression Comparison (FEC), to train the model. Figure 5 shows the model utilized in the proposed approach. The FEC dataset consists of a variety of emotions, including amusement, anger, concentration, disgust, and sadness, among others. The proposed system requires participants to take their facial images weekly using the “Mental Health Protector” application. The facial image is used to extract the participant’s facial expression embedding features.

In this study, we propose sensor domain data-derived features based on depression symptoms. As derived features, we additionally utilize facial expression features extracted from image domains using deep learning. Therefore, we propose multimodal-based derived features: sensor-domain and image-domain. The following are the features we propose: sleep-derived features for “amount of sleep” and “quality of sleep”, physical activity-derived features for “location variance”, “entropy”, and “the amount of physical activity per day”, and facial expressions features. In total, 33 features are derived from a multimodal dataset. Table 3 summarizes the sensor data and derived features used in the proposed system.

## 3. Results

### 3.1. Definition of Depression

The proposed system utilizes two depression severity assessments (PHQ-9 [15] and CESD-R [16]). The results of PHQ-9 and CESD-R represent severity scores for depression symptoms in the last two weeks. The severity of depressive symptoms increases as the score rises. PHQ-9, the depression screening scale, is made up of nine items that correlate to major criteria for diagnosing depression disorders. It has been widely utilized as a measurement tool in previous studies on depression prediction with smartphone sensors. The PHQ-9 response score is between 0 and 27. According to Kroenke’s study [25], depression can be defined if the total PHQ-9 score is 10 or higher. The cut-point of 10 is used to classify the depressive mood of participants. Consequently, 55 participants are classified into a depressed group, whereas 51 are classified as non-depressed. The CESD-R is a tool for measuring the severity of depression representing the symptoms and duration of major depression. The 20 items of the CESD-R assess depressive symptoms in nine different groups as defined by DSM-5. The CESD-R response score is between 0 and 60. According to Lee [17], the optimal cut-point for experimentally showing depression among Koreans, considering their sociocultural background, is 13. Since every participants in the experiment is Korean, we set the cut-point at 13. On this basis, 84 participants were classified to the depressed group, while 22 participants were classified to the non-depressed group.

### 3.2. Experiment

We present the results of predicting depression for 106 participants using PHQ-9 and CESD-R, respectively. For training and test of the depression prediction model, we randomly divided the 106 participants into training and test data at a 3:1 ratio. Consequently, 79 out of a total of 106 data (74.5%) were categorized as training data, while the remaining 27 data (25.5%) were categorized as test data.

#### 3.2.1. Result

In this paper, we utilize “Random Forest”, a machine learning classification model, to predict participants’ depression. Random forest is an ensemble approach of randomly training multiple decision trees, and many studies have shown its effectiveness in predicting depression. In addition, the validity of the derived feature design approach may be verified since Random Forest is interpretable. For implementation, “Random Forest Classifier” provided by scikit-learn is used, and the experiment is conducted by fixing the maximum depth of the tree (max_depth) to 100, and the number of trees (n_estimators) to 1000.

The depression group defined by PHQ-9 (cut-point ≥ 10) is set as the groundtruth for the experiment. The classifier is trained using the extracted features from the data of 79 participants designated as the training set. The experimental results show the prediction accuracy for the test data. To analyze the proposed derived features, the accuracy of depression prediction using only each derived feature is evaluated. The accuracy of prediction for sleep, physical activity, and facial expression features is 55.56%, 59.26%, and 66.67%, respectively. In particular, the newly proposed deep learning-based facial expression features demonstrated the best accuracy. When all derived features are utilized, 74.07% accuracy is generated. Therefore, the complimentary relationship between the derived features is confirmed. Table 4 shows the accuracy of applying Random Forest to each derived feature.

#### 3.2.2. Comparison with Diagnosis

To examine if the proposed method provides valid results for depressed patients, we compare the predictions of the proposed system to the mental disorder diagnosed by the clinician for each participant. The diagnostic records of 27 participants assigned as test data are divided into depressive/non-depressive disorders. As a result, 16 participants were diagnosed with disorders associated with depression, whereas the remaining 11 were diagnosed with diseases other than depression. Table 5 shows the performance based on a comparison between depression prediction and diagnosis using PHQ-9 and CESD-R, respectively.

The proposed system predicts depression in 15 of the 16 depressed participants when CESD-R is used as the ground truth. In contrast, when PHQ-9 is utilized, only 11 out of 16 depressed patients are predicted to have depression. The precision for identifying depression also has a CESD-R of 65.21%, which is higher than PHQ-9. We find that the system utilizing CESD-R has an f1-score of 76.92%, making it an efficient predictor of depression. In addition, we provide comparison results for 27 test data involving patients without depression. As a result of using CESD-R, both precision and recall showed higher performance compared to PHQ-9. The results for the test data also showed that using CESD-R worked effectively for patients with mental disorders.

## 4. Discussion

Our goal was to examine the feasibility of predicting depression using smartphones in patients with mental illness. In order to verify the prediction system, the accuracy of depressed mood prediction was investigated using the collected data of 106 participants with mental health disorders. The results verify our system’s applicability to patients with mental disorders. In addition, an analysis of depression severity assessments (PHQ-9 and CESD-R) is provided.

### 4.1. Analysis

When the accuracy of predicting depression by PHQ-9 (cut-point ≥ 10) was calculated as the designed induction characteristic, 74.07% performance was shown. The accuracy of depression prediction by CESD-R (cut-point ≥ 13) was 77.08%. The PHQ-9 and CESD-R experiments differ in terms of data balance in addition to performance. We hypothesized that the high accuracy of CESD-R is due to a data imbalance because the number of depression/non-depression groups divided based on the cut-point is PHQ-9 (55:51) and CESD-R (84:22), respectively. Therefore, we investigated why questionnaire-based performance differences occurred and which questionnaire is suitable for the proposed system.

We consider that identifying people diagnosed with depression is more important than accurately predicting depression based on questionnaire responses. When smartphone data are collected from the general public, analysis is impossible due to the absence of medical diagnostic information for the participants. However, we recruited participants to collect smartphone data from mental patients. Therefore, the participants we recruited have been diagnosed by clinicians. We focused on examining the relationship between the proposed system and patients diagnosed with depression because the proposed system aims to automatically pre-detect users with signs of depression and recommend treatment. Therefore, it is important for our system to accurately identify depressed patients.

To investigate the relationship between the proposed system’s predictions and diagnosis, we divided the disorders of the participants into depression and non-depression diseases. In the experiment, PHQ-9 and CESD-R were each set as the ground truth, and the predictions and diagnosis were compared. When compared to the diagnosis, the results of CESD-R were convincing. CESD-R predicted that 15 (93.75%) out of 16 participants with depression are depressed. In contrast, PHQ-9 predicted that 11 (68.75%) out of 16 participants are depressed. CESD-R showed optimal performance in detecting patients with depression. In addition, experiments including non-depressed patients for CESD-R provide higher performance, with f1-scores of 61.88%, than PHQ-9. Therefore, we demonstrate that it is efficient to utilize CESD-R in the proposed system to effectively predict a larger number of depressed patients.

### 4.2. Lessons

We collected smartphone data on patients with mental disorders. Using the collected datasets, we demonstrated the feasibility of the proposed depression prediction system. However, there are still some limitations to the practical use of the proposed system. The participants included in the collected data were recruited from a single hospital. In addition, it cannot be assured that the sample size is sufficient. This makes it difficult to generalize our results. Therefore, the general growth of the proposed system requires public data collection. In addition, future studies should collect larger samples.

As it only supports Android-based devices, it is impossible to ensure that it would perform well for iOS-based devices. The “Mental Health Protector” application operates only on Android-based smartphones. While collecting smartphone data, the application should run in the background. According to iOS’s security policy, it is not possible to collect sensor data in the background. Because our system does not support iOS, we were unable to collect data from a significant number of participants. Therefore, it is necessary for future research to develop an iOS-based application so they may collect all data.

In this paper, various multimodal-based passive sensor datasets are collected using the “Mental Health Protector”. However, only GPS, Accelerometer, Gyroscope, Facial Expression Features, and Screen on/off are utilized in the proposed system. We have attempted to use various data in the design of derived features. However, due to unanticipated difficulties with the collected data, it was not possible to extract significant derived features. For example, since the viewpoint of the acquired facial image was not fixed, it was difficult to compare the landmarks precisely. Therefore, it is essential to develop an application with specificity to prevent noise in feature extraction.

## 5. Conclusions

We propose an automated depression prediction system using smartphones. By newly proposing image-based derived features, we propose a multimodal-based derived features design method related to depression. To collect data and predict depression, we construct a system consisting of a smartphone application and a big data-based cloud platform. Based on the established system, multimodal data of 106 participants who visited psychiatry and two self-reported questionnaires were collected. This paper shows the feasibility of depression prediction using data from 106 people collected through the proposed system. In addition, it was shown to be effective for depressed patients by comparing the doctor’s diagnosis with the proposed system’s predictions. In future studies, the accuracy of automated depression prediction may be enhanced by including hospital-visiting patient data into the model as a recommendation by the proposed system.

## Figures and Tables

**Table 1:** Data table list collected from “Mental Health Protector” application. Multimodal sensor data collected for each user are transmitted to a cloud-based big data platform according to a predefined table. Data information about table names can be found in the description.

Number	Table Name	Description
1	TB_WEB_USR_INFO	User’s web information
2	TB_API_USER_INFO	API user information
3	CALL_LOGS	Call incoming/outgoing history
4	SMS_LOGS	SMS incoming/outgoing history
5	CELL_INFO	Network signal level and quality information
6	BATTERY_INFO	Battery power level data for user’s device
7	SENSORS	Sensor data for user’s device
8	BLUETOOTH_DEVICES	Bluetooth device data near user’s device
9	LOCATIONS	GPS for user’s device
10	WIFI_INFO	Wi-fi data near user’s device
11	SCREEN_ONOFF	Screen on/off data for user’s device
12	FACE_LANDMARK	3D facial landmarks coordinate and expression feature
13	ACTIVITY_TRANSITION	The type of behavior of user
14	BLUETOOTH_TYPE_MAP	Bluetooth device type mapping table
15	SENSOR_MAP	Sensor type mapping table

**Table 2:** Participants’ Demographics. This shows the distribution of demographic information and responses to self-report questionnaires for the participants. Statistics for participants on “age”, “gender”, “depression severity for response scores”, and “depressed mood by cut-point” are provided.

	Variable	Value, n(%)
Gender	Male	43 (40.57)
	Female	63 (59.43)
Year of birth	1960	4 (3.77)
	1965	6 (5.66)
	1970	13 (12.26)
	1975	17 (16.04)
	1980	14 (13.21)
	1985	7 (6.60)
	1990	14 (13.21)
	1995	15 (14.15)
	2000	16 (15.09)
Depression severity (PHQ-9)	severe	5 (4.72)
	moderately severe	8 (7.55)
	moderate	20 (18.87)
	minor	22 (20.75)
	minimal	31 (29.25)
	normal	20 (18.87)
Depressed mood (CESD-R)	Depressive	84 (79.25)
	Non-Depressive	22 (20.75)

**Table 3:** Multimodal Derived Features. We present multimodal derived features for predicting depression in participants. For the proposed derived features, screen on/off, GPS, accelerometer, gyroscope, and facial image are utilized. Consequently, 33 features are designed to predict depression.

Derived Feature	Sensor Data	Features
Amount of sleep	Screen on/off	Statistical features of estimated sleep time per day (maximum, minimum, mean, SD, Q1, Q2, Q3)
Quality of sleep		Statistical features of smartphone usage (maximum, minimum, mean, SD, Q1, Q2, Q3)
Location variance	GPS	$\log\left(\sigma_{lat}^{2}+\sigma_{log}^{2}\right)$
Entropy		$-\sum_{i}p_{i}\log p_{i}$
Physical activity per day	Accelerometer, Gyroscope	Average daily physical activity time via Google Activity Recognition Transition API
Facial expression	Facial image	Facial expression features

**Table 4:** Accuracy by Derived Features. The table shows the accuracy of the test data for each derived feature we designed. When all the derived features are combined, 74.07% accuracy is obtained.

	Derived Feature	Accuracy
Sleep	Amount of sleep	55.56
	Quality of sleep	
Activity	Location Variance	59.26
	Entropy	
	Activity	
Facial Expression	Facial Expression	66.67
Total Derived Features		**74.07**

**Table 5:** Random Forest Classification Result Compared to Diagnosis. The table shows the result of comparing the participant’s diagnosis with depression predictions. The “Depression” row represents the performance predicted to be depressing for 16 depressed patients. When CESD-R is used for depressed patients, the recall rate is 93.75% and the precision rate is 65.21%. Therefore, it shows that CESD-R is more effective than PHQ-9 in predicting depression in patients with depression.

	Survey	Precision	Recall	F1-Score
Depression	PHQ-9	64.71	68.75	66.67
	CESD-R	**65.21**	**93.75**	**76.92**
Total	PHQ-9	58.71	59.26	58.91
	CESD-R	**69.20**	**66.67**	**61.88**

## Data Availability

The data are not publicly available due to ethical reasons.

## References

[1] Santomauro D.F., Herrera A.M., Shadid J., Zheng P., Ashbaugh C., Pigott D.M., Abbafati C., Adolph C., Amlag J.O., Aravkin A.Y. (2021). Global prevalence and burden of depressive and anxiety disorders in 204 countries and territories in 2020 due to the COVID-19 pandemic. Lancet.

[2] Bahk Y.C., Park K.H., Kim N.E., Lee J.H., Cho S.R., Jang J.H., Jung D.W., Chang E.J., Choi K.H. (2020). Psychological Impact of COVID-19 in South Korea: A Preliminary Study. Korean J. Clin. Psychol..

[3] Li L., Li F., Fortunati F., Krystal J.H. (2020). Association of a Prior Psychiatric Diagnosis With Mortality Among Hospitalized Patients With Coronavirus Disease 2019 (COVID-19) Infection. JAMA Netw. Open.

[4] US Centers for Disease Control and Prevention People with Certain Medical Conditions. 25 February 2022. https://www.cdc.gov/coronavirus/2019-ncov/need-extra-precautions/people-with-medical-conditions.html.

[5] American Psychiatric Association (2013). Diagnostic and Statistical Manual of Mental Disorders.

[6] Wang R., Chen F., Chen Z., Li T., Harari G., Tignor S., Zhou X., Ben-Zeev D., Campbell A.T. StudentLife: Assessing mental health, academic performance and behavioral trends of college students using smartphones. Proceedings of the 2014 ACM International Joint Conference on Pervasive and Ubiquitous Computing.

[7] Colbaugh R., Glass K., Global V. (2020). Detecting and monitoring brain disorders using smartphones and machine learning. medRxiv.

[8] Ware S., Yue C., Morillo R., Lu J., Shang C., Bi J., Kamath J., Russell A., Bamis A., Wang B. (2020). Predicting depressive symptoms using smartphone data. Smart Health.

[9] Opoku Asare K., Terhorst Y., Vega J., Peltonen E., Lagerspetz E., Ferreira D. (2021). Predicting Depression From Smartphone Behavioral Markers Using Machine Learning Methods, Hyperparameter Optimization, and Feature Importance Analysis: Exploratory Study. JMIR Mhealth Uhealth.

[10] Saeb S., Lattie E., Kording K., Mohr D. (2017). Mobile Phone Detection of Semantic Location and Its Relationship to Depression and Anxiety. JMIR Mhealth Uhealth.

[11] Narziev N., Goh H., Toshnazarov K., Lee S.A., Chung K.-M., Noh Y. (2020). STDD: Short-Term Depression Detection with Passive Sensing. Sensors.

[12] Wang R., Wang W., daSilva A., Huckins J.F., Kelley W.M., Heatherton T.F., Campbell A.T. Tracking Depression Dynamics in College Students Using Mobile Phone and Wearable Sensing. Proceedings of the ACM on Interactive, Mobile, Wearable and Ubiquitous Technologies.

[13] Doryab A., Min J.K., Wiese J., Zimmerman J., Hong J. Detection of behavior change in people with depression. Proceedings of the Workshops at the Twenty-Eighth AAAI Conference on Artificial Intelligence.

[14] Dang M., Mielke C., Diehl A., Haux R. (2016). Accompanying depression with FINE—A smartphone-based approach. Stud. Health Technol. Infor..

[15] Kroenke K., Spitzer R.L., Williams J.B. (2001). The PHQ-9: Validity of a brief depression severity measure. J. Gen. Intern. Med..

[16] Eaton W.W., Smith C., Ybarra M., Muntaner C., Tien A. (2004). Center for Epidemiologic Studies Depression Scale: Review and Revision (CESD and CESD-R). Use Psychol. Test. Treat. Plan. Outcomes Assess. Instrum. Adults.

[17] Lee S., Oh S.T., Ryu S.Y., Jun J.Y., Lee K.S., Lee E., Park J.Y., Yi S.W., Choi W.J. (2016). Validation of the Korean version of Center for Epidemiologic Studies Depression Scale-Revised(K-CESD-R). Korean J. Psychosom. Med..

[18] Cuttone A., Bækgaard P., Sekara V., Jonsson H., Larsen J.E., Lehmann S. (2017). SensibleSleep: A Bayesian model for learning sleep patterns from smartphone events. PLoS ONE.

[19] Chen C.-Y., Vhaduri S., Poellabauer C. Estimating Sleep Duration from Temporal Factors, Daily Activities, and Smartphone Use. Proceedings of the IEEE 44th Annual Computers, Software, and Applications Conference (COMPSAC).

[20] Min J.-K., Doryab A., Wiese J., Amini S., Zimmerman J., Hong J.I. Toss ‘n’ turn: Smartphone as sleep and sleep quality detector. Proceedings of the ACM Conference on Human Factors in Computing Systems.

[21] Sano A., Taylor S., McHill A., Phillips A., Barger L., Klerman E., Picard R. (2018). Identifying Objective Physiological Markers and Modifiable Behaviors for Self-Reported Stress and Mental Health Status Using Wearable Sensors and Mobile Phones: Observational Study. J. Med. Internet Res..

[22] Saeb S., Zhang M., Karr C., Schueller S., Corden M., Kording K., Mohr D. (2015). Mobile Phone Sensor Correlates of Depressive Symptom Severity in Daily-Life Behavior: An Exploratory Study. J. Med. Internet Res..

[23] Zhou X., Jin K., Shang Y., Guo G. (2020). Visually Interpretable Representation Learning for Depression Recognition from Facial Images. IEEE Trans. Affect. Comput..

[24] Vemulapalli R., Aseem A. A Compact Embedding for Facial Expression Similarity. Proceedings of the IEEE Conference on Computer Vision and Pattern Recognition.

[25] Costa M.V., Diniz M.F., Nascimento K.K., Pereira K.S., Dias N.S., Malloy-Diniz L.F., Diniz B.S. (2016). Accuracy of three depression screening scales to diagnose major depressive episodes in older adults without neurocognitive disorders. Rev. Bras. Psiquiatr..

